# Beneficial Effects of Olive Oil Enriched with Lycopene on the Plasma Antioxidant and Anti-Inflammatory Profile of Hypercholesterolemic Patients

**DOI:** 10.3390/antiox12071458

**Published:** 2023-07-20

**Authors:** Jesus Roman Martínez Álvarez, Ana Belen Lopez Jaen, Monica Cavia-Saiz, Pilar Muñiz, Victoria Valls-Belles

**Affiliations:** 1 Departamento de Enfermería, Escuela Universitaria de Enfermería, Fisioterapia y Podología, Universidad Complutense, 28040 Madrid, Spain; jrmartin@med.ucm.es; 2 Departamento de Biotecnología, Universidad de Alicante, 03690 Alicante, Spain; ana.belen.lopez@ua.es; 3 Departamento de Biotecnología y Ciencia de los Alimentos, Universidad de Burgos, 09001 Burgos, Spain; monicacs@ubu.es (M.C.-S.); pmuniz@ubu.es (P.M.); 4 Unidad Predepartamental de Medicina, Facultad de Ciencias de la Salud, Universidad Jaume I, 12006 Castellón, Spain

**Keywords:** lycopene, olive oil, CRP, cholesterol, cytokines, antioxidant capacity, oxidative stress biomarkers

## Abstract

Olive oil and lycopene are foods that have potent antioxidant activity. The objective was to determine the effects of consumption of olive oil enriched with lycopene on oxidative stress biomarkers in hypercholesterolemic subjects. We examined the effects of oil enriched with lycopene extract daily intake during 1 month on plasma antioxidant capacity, lipids profile (triacylgycerols, total cholesterol, cHDL; cLDL, ox-LDL), biomarkers of oxidative stress, and inflammatory markers related with atherosclerosis risk (C-reactive protein (CRP), IL-6; sDC4L) in subjects hypercholesteremics (cholesterol > 220 mg/dL). In the group consuming olive oil-lycopene, significant increases (*p* < 0.05) in the levels of plasma lycopene concentration (0.146 ± 0.03 versus 0.202 ± 0.04 (µmol/L)), α-carotene (0.166 ± 0.064 versus 0.238 ± 0.07) and in β-carotene (0.493 ± 0.187 versus 0.713 ± 0.221) were observed. These results are linked with the increases of plasma antioxidants and decreases biomarkers of oxidative stress (carbonyl groups, malondialdehyde and 8-hydroxy-deoxiguanosine) observed in hypercholesterolemic group. In relation to lipid profile, a significant decrease was observed in the levels of ox-LDL (781 ± 302 versus 494 ± 200), remaining unchanged the levels of TG, cholesterol, HDL and LDL-c. Regarding inflammatory biomarkers, the levels of CRP and IL-6 decreased significantly. The positive results obtained in this study support the use of olive oil enriched with lycopene to reduce the risk of coronary disease.

## 1. Introduction

Atherosclerosis is a chronic inflammatory disease that is responsible for several cardiovascular events such as ischemic heart disease, coronary artery disease, peripheral artery disease, and ischemic stroke [1]. The prevalence of atherosclerosis is increasing worldwide and due to the aging population, the incidence of cardiovascular diseases is expected to rise, together with disease-related deaths and disabilities [2]. Factors as unhealthy nutrition, physical inactivity, hypercholesterolemia, hyperglycemia, smoking, etc. influence the occurrence of cardiovascular disease.

The mechanisms by which hyperlipidaemia contributes to atherogenesis include particularly hypercholesterolemia, inflammation and oxidative modifications of LDL and cholesterol by reactive oxygen species (ROS) generated in macrophages or endothelial cells. Once in the subendothelial space LDL-C undergo an oxidation thus making them por-inflammatory and activating the overlying endothelium [3]. The inflammation is the underlying cause of many of the risk factors associated with heart disease, such as high blood pressure, high cholesterol and obesity [1,4]. The early phase of atherosclerosis starts with endothelial dysfunction which undergo an inflammatory response. Furthermore, the inflammation-induced endothelial dysfunction results in an increased permeability to lipoproteins and their subendothelial accumulation, leukocyte recruitment and platelets activation [3].

Moreover, ROS (reactive oxygen species) play a key role in the pathogenesis of atherosclerosis; large amounts can overwhelm the antioxidant defence, causing lipid peroxidation, protein modification, and DNA oxidation. Increasing evidence suggests that lycopene is able to modulate redox status through different mechanisms such as interaction with ROS, ROS-producing enzymes modulating, and the control of redox-sensitive molecular pathways [5,6].

Therefore, the control of systemic inflammation is important in preventing cardiovascular diseases. A healthy diet, regular exercise and stress reduction can all help to reduce inflammation and prevent chronic diseases [1]. A significant number of studies indicate that the Mediterranean diet is associated with preventive effect of several chronic diseases that are either directly or indirectly related to inflammation. Thus, the intake of fruits and vegetables diminishes the prevalence of this cardiovascular diseases [7]. Tomato is a product present in the Mediterranean diet, which has been associated with a low mortality from cardiovascular disease [8,9,10]. In the food industry as result of the overproduction of fresh tomatoes or losses during processing (juices, sauces, etc) generate high amount of waste which is rich in the bioactive compound lycopene [8]. On the other hand, the olive oil is considered an excellent source of lipids, being associated with the primary and secondary prevention of cardiovascular disease to improvement of the lipid profile and increased oxidative satiability [11].

The amount of lycopene in tomatoes is about 0.72–20 mg lycopene per 100 g. In addition to the amount of lycopene present in a food, it is important to know the bioavailability that can be affected by different factors such as amount of lycopene consumed in meal, food matrix, co-ingestion of fat or effects of absorption. Therefore, Petyaev et al. (2016) indicate that the average of consume of lycopene for European population is in the ranges of 5 to 7 mg/day and for United States is among 3.7–16.1 mg [12]. Furthermore, the low toxicity and high tolerance of lycopene is used for the design of lycopene supplementation protocols. The beneficial effects of lycopene to prevent the atherogenesis is attributed to its capacity modulate oxidative stress and cytokine activation though its antioxidant properties [10,13].

Several epidemiological studies have shown an inverse relationship between the intake of lycopene, levels in serum, and the incidence of cardiovascular diseases [14,15] and that this effect was comparable to the one by fluvastatin [16]. Lycopene preventive effect against cardiovascular disease by their ability to modulate inflammation to reduce the LDL-induced foam cells formation, decreasing lipid synthesis [17], inhibiting TNF-alpha, induced activation of NF-kB [18]. Furthermore, lycopene is a highly effective antioxidant to scavenger of singlet oxygen and the reduction of reactive oxygen species such as hydrogen peroxide, nitrogen dioxide and hydroxyl radicals [5]. As antioxidant lycopene shows cardioprotective effect to prevent against oxidative stress-induced myocardial hypertrophy por improving ROS production, inhibition of LDL oxidative damage and improving endothelial function [6]. Moreover, the bioavailable of lycopene depend of their structure or the interaction with other compounds. Researches confirm that bioavailability of lycopene is higher when consumed as a sauce prepared with a fat base such as olive oil than when it is consumed in natural fruit or juice.

The bioactive compounds of olive oil have anti-inflammatory and antiteratogenic activity, improving the lipid profile and reducing oxidative stress reducing of mortality associated with cardiovascular diseases [11]. One of these bioactive compounds are the phenolic compound which act as antioxidants preventing and reducing events of cardiovascular disease by means of the inhibition of lipid peroxidation caused by ROS an inhibit the activity of LDL-c. The content of MUFAs, especially oleic acid is also associated with the protective effect against cardiovascular disease [19].

On the basis of the before mentioned, the purpose of this work is to study the effect of lycopene-enriched olive oil on lipid profile and markers of inflammation in people with moderate non-familial hypercholesterolemia without pharmacological treatment.

## 2. Materials and Methods

### 2.1. Lycopene Product

The study product sample “lycopene-olive oil” consisted of virgin olive oil enriched with lycopene (350 mg/L). The olive oil samples with licopene used were “Aceisterol^®^” (extra ecologic virgin olive oil, obtained using olives of the Morisca variety, enriched with a tomato extract, its oleoresin natural extract without additives with high lycopene content), free of charge for the study. All samples belonged to the same production batch of 20 mL single-dose units.

### 2.2. Study Population and Study Design

Patients (n = 80) were recruited from the town of Moralzarzal, Madrid, Spain, including healthy men and woman with cholesterol levels over 220 mg/dL. Exclusion criteria included familial hypercholesterolemia, and the use of any medication affecting blood lipids or the intake of vitamin or mineral supplements. The protocol was approved by the Etic Committee/Teaching Unit of the “Collado Villalba Pueblo” Healthcare Centre, and informed consent was obtained from volunteers to participate. The design of this nutritional intervention study was randomized, and the subjects were allocated to one of two groups: (1) supplemented group (n = 40) who consumed 40 mL/day (20 mL at lunch and 20 mL at dinner) of an olive oil enriched with lycopene (7 mg/20 mL); and, (2) control group (n = 40) who consumed 40 mL/day of standard olive oil. Both groups consumed the daily study dose of oil for 1 month, in addition to their usual diet, with the proportions of 34% of energy as carbohydrates, 20% proteins, and 46% as fats (34% saturated fatty acids, 19.7% monounsaturated fatty acids, and 46.3% polyunsaturated fatty acids).

### 2.3. Blood Samples

Blood samples for study were collected at the beginning of the study (initial value) and after 1 month of olive oil enriched with lycopene intake. Plasma was separated by centrifugation (3000 g, 10 min) and stored at −80 °C.

### 2.4. Plasma Carotenoids Assays

Plasma lycopene, carotenes, and cryptoxanthin concentrations were analysed by using a high-pressure liquid chromatographic modified by Talwar, Ha, Cooney, Brownlee, O’Reilly [20]. The plasma was obtained from heparinized tubes (5% sodium heparin). After, the plasmas diluted 1:6 with water, and after 70 of ethanol and ascorbic acid 0.01% and 2 mL of hexane were added and centrifuged to 2500 rpm 20 min. Hexane was dried under nitrogen and the sample was reconstituted with tetrahydrofuran and analyzed by using a HPLC Gilson with a Waters C30 column YMC^TM^ Carotenoid 5 µm (4.6 × 250 mm) with a mobile phase of methanol-acetonitrile-tetrahydrofuran of 80/40 to 10/90 in gradient. A UV/V detector (Kontron Instruments, Madrid, Spain) set at a 450 nm wavelength was used.

### 2.5. Total Antioxidant Capacity

ABTS method was determined following the ABTS (2, 2′-azino-bis-[3-ethylbenzothiazoline-6-sulfonic acid]) method [21]. Briefly, the ABTS^+^ radical cation is generated by the reaction of a 7 mM solution of ABTS in water with 2.45 mM potassium persulphate (1:1). The assay is made up with 980 μL of ABTS^+^ and 20 μL of the oil sample (at a dilution 1:200) or 40 μL of plasma, measuring the inhibition of the absorbance of the ABTS^+^ cation at 734 nm caused by the action of the oil sample. The results are expressed as molar equivalents (mM) of trolox using a calibration curve.

FRAP method was employed to measure the reduction capacity of samples [21]. This method measures the increase of absorbance at 593 nm due to the formation of tripyridyl-s-trizine complexes with ferric (II) (TPTZ-Fe (II)) in the presence of a reductive agent. The FRAP reagent was prepared by mixing 25 mL of sodium acetate 0.3 M, 2.5 mL TPTZ (10 mM), 2.5 mL of FeCl_3_ 20 mM and 3 mL of water. 20 μL of oil was added to the FRAP reagent and the mixture was incubated at 37 °C monitoring the reaction up to 30 min. The results are expressed as molar (mM) equivalents of Fe (II) using a calibration curve.

DPPH method was determined following the method of Brand-Williams [22], using the 2.2-stable radical diphenil-1-picrylhydrazyl (DPPH) which has its absorption maximum at 517 nm. A 980 μL of 60 mM DPPH was added 20 μL of oil. Incubated at room temperature for 2 h, and evaluated spectrophotometrically at 517 nm. The results are expressed as millimoles of Trolox using a calibration curve with different concentrations of Trolox.

Hydroxyl scavenging activity, was determined following the methodology given by Halliwel et al. [23]. Briefly, different 50 μL of sample (plasma or oil) were incubated at 37 °C for 1 h in a final volume of 1 mL containing the following reagents at a final concentration stated: phosphate buffer (50 mM, pH: 7.4), deoxyribose (10 mM), ascorbic acid (1 mM) and copper (II) ions (100 μM). Then, 1 mL of the reaction solution was mixed with 0.5 mL of 28% (*w*/*v*) of trichloroacetic acid (TCA) and 0.5 mL of thiobarbituric acid (TBA, 1% *w*/*v*, in 0.05 M NaOH) solutions, heated at 100 °C for 15 min, then cooled at room temperature. The amount of color formation, generated during the incubation time before the addition of TBA-TCA, was spectrophotometrically measured at 532 nm. An inhibition in the color formation results from the scavenging of hydroxyl radicals by the presence of compounds with antiradical capacity. Results are expressed as the inhibition (expressed as a percentage, %) caused on the deoxyribose degradation by juices when compared with mixtures containing blank samples.

Superoxide scavenging activity, determination was carried out following the methodology proposed by Liu [24]. Briefly, radicals are generated in a phenazin methosulfate-β-nicotinamide adenine dinucleotide system by oxidation of NADH and assayed by the reduction of nitroblue tetrazolium (NBT). In this experiment, the superoxide radicals were generated in Tris-HCl buffer (16 mM, pH: 8.0) which contained 78 μM NADH, 50 μM NBT, 10 μM PMS and 200 μL of olive oil was used. The colour reaction between superoxide radicals and NBT was detected at OD 560 nm.

### 2.6. Biomarkers of Oxidative Stress

Malondialdehyde (MDA) levels were determined in plasma according to the method of Santos et al. [25] by the use of HPLC. Briefly, samples were hydrolyzed with 1 mL 100% TCA (*w*/*v* in 0.6 N HCl), 0.2 mL 0.12 M TBA (*w*/*v* in 0.26 M Tris-ClH). This leads to formation of a complex with thiobarbituric acid, which was determined at 532 nm.

Carbonyl group (GC), protein oxidation biomarker, was measured by an estimation of carbonyl groups released during the incubation time using the method of Levine et al. [26]. The samples were centrifuged at 13,000 rpm for 10 min. Following, 20 µL of this plasma was placed in a 1.5 mL eppendorf, and 400 µL of 10 mM 2,4 dinitrophenylhydrazine (DNFH)/2.5 M ClH and 400 µL of 2.5 M ClH to were added the control samples. This mixture was incubated for 1 h at room temperature. Protein precipitation was performed using with 1 mL of 100% of TCA, washed twice with ethanol/ethyl acetate (1:1 *v*/*v*), and samples were centrifuged at 12,600 rpm for 3 min. Finally, 1.5 mL of 6 N guanidine, pH 2.3, was added and the samples were incubated in a 37 °C water bath for 30 min and were centrifuged at 12,600 rpm for 3 min. Protein concentration was spectrophotometrically calculated measuring the absorbance of the sample at 373 nm, using a molar absorption coefficient of 22,000 M^−1^cm^−1^. Results are expressed as nmol/mg protein.

8-OHdG levels, was measured in DNA extracted from the samples by proteinase K digestion in combination with DNeasy Blood & Tissue kit (QIAGEN, Hilden, Germany). Briefly, 0.5 μg DNA/μL of samples were incubated with 100 units of DNase I in 40 μL 10 mM Tris–HCl at 37 °C for 1 h. The pH of the reaction mixture was then lowered with 15 μL of 0.5 M sodium acetate (pH 5.1), 10 μL of nuclease P1 (five units) and incubated for 1 h. Readjustment of pH with 100 μL of 0.4 M Tris–HCl (pH 7.8) was followed by the addition of three units of alkaline phosphatase; samples were incubated for longer than an hour. Analysis of 8-OHdG was determined by ELISA using the kit 8-OH-dG-EIA-Biotech (Oxis Health Products Inc., Portland, OR, USA).

### 2.7. Plasma Lipid Profile

Lipid measurements were performed using the autoanalyser MODULAR EDDPP, ROCHE. Triglycerides, total cholesterol, HDL-c and LDL-c were determined by commercial enzymatic tests (Roche Diagnostics GMBH, Holzheim, Germany). Auto-antibodies against oxidized LDL (oxLDL antibodies) were detected in serum using an enzyme immunoassay Biomedica Medizinprodukte (GMBH and Co KGA-1210, Wien, Autriche).

### 2.8. Inflammatory Markers Assay

PCR were performed using the COBAS INTEGRA 800 auto analyser of ROCHE. Determination of sCD40L and IL-6 was performed in plasma using Diaclone Research ELISA kits, according to the manufacturer’s instructions.

### 2.9. Statistical Analysis

The sample size was calculated considering that to reduce around 10% total cholesterol, with a 10% error probability and a power of 80% using the paired student *t* test, 40 patients are needed. Results are presented as mean standard deviation and a *p* < 0.05 was considered statistically significant.

## 3. Results and Discussion

The lycopene-oil used for this study was obtained from extra ecologic virgin olive oil, obtained using olives of the Morisca variety, enriched with the lycopene contained in a tomato extract. The composition of the sample contained in the 20 mL single-dose units is shown in Supplemented Table S1. The concentration of lycopene in the oil was of 0.05%. Regarding fatty acids, oleic acid content represents 71.62 %, 11.96 % palmitic acid, 9.29% linoleic acid, 3.24% stearic acid, and 1.07% linolenic acid.

Hypercholesterolemic subjects (n = 80) were randomized to receive olive oil enriched with (lycopene-olive oil) (n = 40) or olive oil (n = 40) as control group. The characteristics of the subjects is shown in Supplemented Table S2.

**Figure 1:**
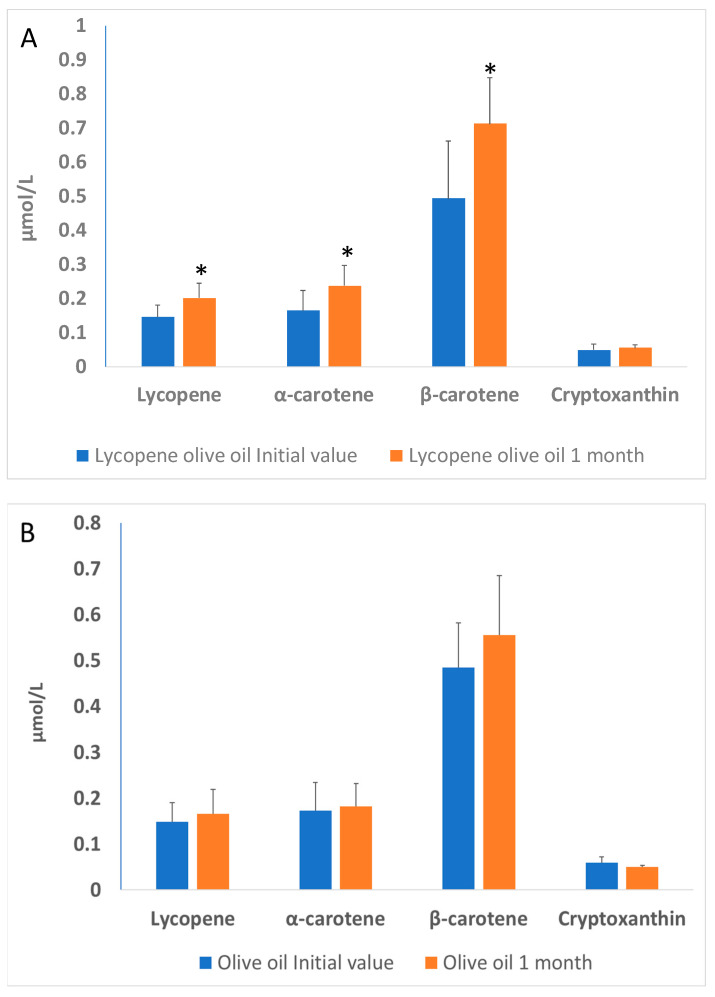
(**A**)—Lycopene, α-carotene, β-carotene and cryptoxanthin levels in the plasma of patients with hypercholesterolemia, at the basal time (initial value) and after 30 days of diet supplementation with lycopene-olive oil (**A**) and olive oil (**B**). Values are means ± SD. Different alphabetical letters indicate significant differences (*p* < 0.005). * Indicates that significant different (*p* < 0.05) between initial value and 1 month.

Blood samples were collected the day before supplementation (initial) and the day after the supplementation period (1 month). The increase in plasma after one month intake (Figure 1A) demonstrates the lycopene bioavailability in patients whose diet was supplemented with lycopene-olive oil. The consumption of lycopene-enriched oil for one month resulted in a significant increase (*p* < 0.05) of 27.2% in lycopene compared to the initial measurement, while the olive oil group did not show significant differences. Additionally, lycopene-oil supplementation led to a 30.2% increase in α-carotene and a 32.8% increase in β-carotene levels, with no significant change in the levels of cryptoxanthin.

The olive oil group (Figure 1B) did not show significant differences between the initial time and 1-month olive oil supplementation. These results are consistent with those observed by other authors, indicating a significant increase in both carotenoids and lycopene. For instance, Porrini et al. [27] demonstrated in a study with women that the consumption of 25 g of tomato puree (containing 7 mg of lycopene and 0.3 mg of β-carotene) for 14 days resulted in an increase of lycopene and β-carotene in lymphocytes and plasma. In a study conducted by Nisnhhimura et al. [28] a daily consumption of 8 mg of lycopene for 12 weeks resulted in a significant increase in plasma lycopene levels compared to a placebo group [22,23,24,25,26,27].

When studying the effect of lycopene-oil intake on plasma antioxidant capacity, we observed a beneficial effect of consuming lycopene-olive oil and olive oil on oxidative stress, as shown in Table 1. The intake of lycopene-olive oil and olive oil did not alter the total antioxidant capacity measured as ABTS, but there was a significant increase (*p* < 0.05) in hydroxyl radical scavenger activity (HRSA) in both groups. We used the ABTS method to measure the synergy of different plasma antioxidants in stabilizing the chemical radical ABTS, and the hydroxyl radical activity (HRSA) method to estimate the plasma capacity to neutralize excessive hydroxyl radical formation, which is highly toxic in biological systems. These findings are consistent with earlier studies [29] where it was observed that the intake of tomato puree for three weeks did not significantly affect the total antioxidant capacity of the plasma. However, a positive relationship between lycopene levels and plasma total antioxidant capacity was observed. On the contrary, Mackinnon et al. [30] detected a significant increase in total antioxidant capacity measured by the ABTS method in postmenopausal women after consuming lycopene-rich tomatoes or lycopene capsules for four months. Also, an increase in HRSA was observed in the group that consumed olive oil alone. This result suggests a role of olive oil in this marker. The hydroxyl scavenger capacity of lycopene-olive oil and olive oil is attributed to the bioavailability of phenolic compounds in the oil and lycopene [11,31]. Therefore, the observed antioxidant capacity and HRSA activity may contribute to the prevention of cardiovascular events caused by reactive oxygen species (ROS). The phenolic compounds and lycopene help suppress the hydroxyl radical and interrupt the oxidation of LDL-C.

**Figure 2:**
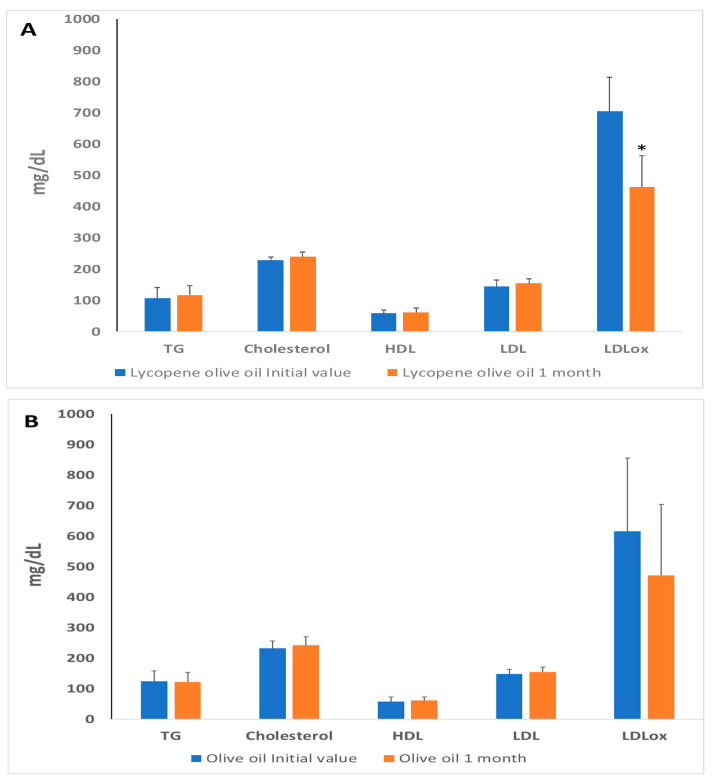
Blood lipid profile levels in plasma of patients with hypercholesterolemic, at basal time (initial value) and after 30 days of diet supplementation with lycopene-olive oil (**A**) and olive oil (**B**). TG = triglycerides; HDL = high-density lipoprotein; LDL: low-density lipoprotein; LDLox: LDL oxidized low-density protein. Values are means ± SD. * Indicate that after 1 month the means are significantly different (*p* < 0.05) when compared to initial time values.

Plasma cholesterol levels, including LDL-C, HDL-C, and serum triglycerides, are commonly used as biomarkers for assessing lipid profiles [32]. It is known that LDL oxidation is a risk factor for heart diseases, and its increased presence plays a key role in the development of atherosclerosis. Oxidized LDL is not recognized by its receptor, leading to its phagocytosis by macrophages and the formation of foam cells, triggering an inflammatory response in endothelial cells and contributing to the progression of the atherosclerotic process. Figure 2 displays the initial values and lipid profiles after a 1-month study for both the olive-lycopene supplemented group and the oil group (Figure 2A). The results indicate that triglyceride, cholesterol, LDL, and HDL levels did not change significantly. However, ox-LDL levels decreased significantly only in the group supplemented with lycopene-olive oil. Our study demonstrates that lycopene-olive oil supplementation resulted in a reduction of oxidized LDL levels compared to the initial values, in contrast to the control group consuming only olive oil (Figure 2B).

Some authors argue that controlling the activities of inflammatory mediators through diet, exercise, and a healthy lifestyle can reduce the incidence of cardiovascular diseases [1,4]. It is well-established that inflammation is considered the main cause of coronary heart disease. The beneficial impact of lycopene and olive oil on cardiovascular risk prevention is primarily attributed to the improvement of endothelial function and the reduction of inflammatory mediators. Therefore, the consumption of lycopene and olive oil plays a significant role in inhibiting the inflammatory response, which is closely associated with the suppression of reactive oxygen species (ROS). An inverse correlation between serum carotenoids and inflammatory biomarkers such as CRP or IL-6 has been observed in relation to cardiovascular diseases [33,34]. The effects of oil intake on inflammation markers, including C-reactive protein (CRP), soluble CD40 ligand (sCD40L), and interleukin 6 (IL-6), are shown in Table 2. The results are presented as a percentage relative to the initial time. The findings reveal a significant decrease in CRP and IL-6 markers in subjects consuming lycopene-olive oil, while no significant changes were observed in the control group (olive oil). However, the levels of sCD40L, another marker, remained unchanged in subjects consuming lycopene-olive oil and significantly increased in the control group. These results highlight the anti-inflammatory properties of lycopene as observed in patients with hypercholesterolemia. These findings are consistent with other studies demonstrating the anti-inflammatory effects of lycopene, which are associated with the inhibition of proinflammatory molecules such as CRP and IL-6 [18,35].

**Figure 3:**
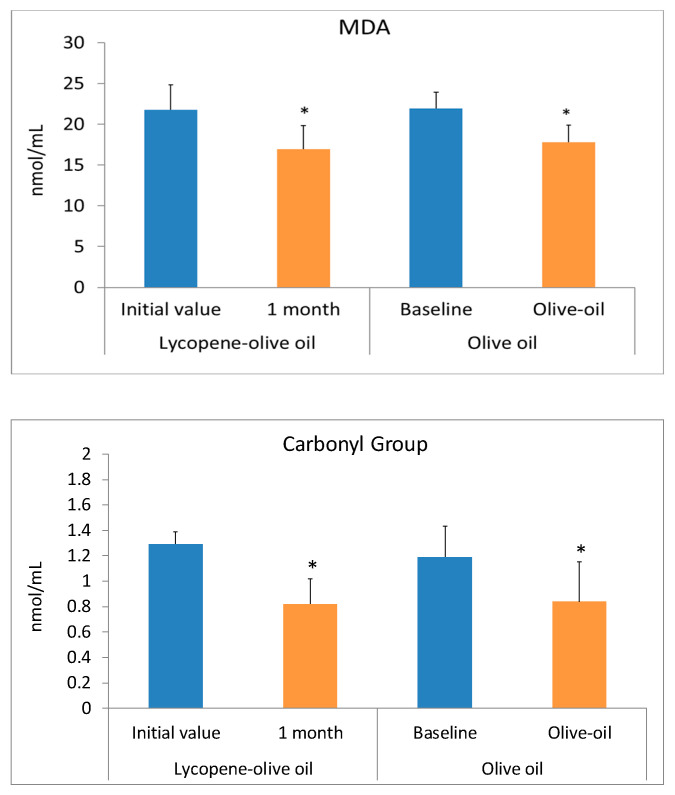

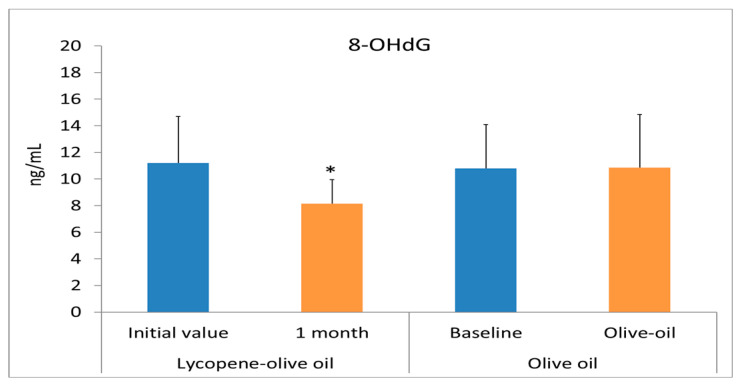
Biomarkers of oxidative stress (MDA, Carbonyl groups, and 8OHdG) levels in plasma of patients with hypercholesterolemic, at basal time (initial value) and after 1 month of diet supplementation with lycopene-olive oil and olive oil. Values are means ± SD. * Indicate that means values after 1 month are significantly different (*p* < 0.05) when compared to initial time baseline.

Oxidative stress is a process characterized by an increase in the oxidant state and a reduction in antioxidants. Consumption of antioxidant and anti-inflammatory compounds can serve as a preventive method for cardiovascular diseases. Hypercholesterolemic individuals suffer from high oxidative stress, as evidenced by elevated biomolecular damage. Our results suggest that consuming lycopene-olive oil can reduce this biomolecular damage. The health benefits of olive oil and lycopene are well-known and result from the presence of bioactive compounds in both substances [13,33,36,37,38]. All plasma biomarkers of oxidative stress, including MDA, GC, and 8OHdG, significantly decreased after one month of lycopene-oil intake (Figure 3). In the control group consuming only olive oil, a significant decrease was also observed, but the values were quantitatively lower compared to the lycopene-olive oil group. The lower MDA levels observed in the lycopene-olive oil group support findings from other studies showing that lycopene can reduce lipid peroxidation [39]. Notably, there was a substantial reduction in oxidative DNA damage, as evidenced by the 8-OHdG levels in hypercholesterolemic patients after one month of lycopene-olive oil intake (27.2% reduction). The DNA protection provided by lycopene is well-documented and is reflected in its ability to defend against DNA damage resulting from reactive oxygen species (ROS) aggression. [40].

Therefore, our study suggests that lycopene-olive oil can be used as a supplement to reduce oxidative stress and the inflammatory process in hypercholesterolemic subjects, thereby potentially lowering the risk of atherosclerosis. This preventive effect is associated with the increased antioxidant and scavenging capacity observed in plasma after supplementation with lycopene-olive oil.

## 4. Conclusions

In conclusion, this study suggests that foods containing olive oil and lycopene can significantly contribute to reducing LDL-induced oxidative stress. This is achieved by increasing endogenous carotenoid levels, which enhance the antioxidant status, and decreasing inflammation-related parameters associated with cardiovascular diseases. Therefore, this dietary combination contributes to the beneficial effect of preventing atherosclerotic disease

## Figures and Tables

**Table 1:** Effect of lycopene-olive oil consumption on plasma total antioxidant capacity in hypercholesterolemic patients.

	Lycopene-Olive Oil		Olive Oil	
	Initial	One Month	Initial	One Month
ABTS (mM)	4.12 ± 0.45	4.1 ± 0.76	4.34 ± 0.16	4.12 ± 0.52
HRSA (% inhibition)	55.8 ± 8.6	64.6 ± 7.7 *	59.0 ± 8.6	65.4 ± 6.7 *

Data presented are means ± SD. * Indicates significant differences between initial and one month values.

**Table 2:** Plasma inflammation markers in hypercholesterolemic patients at the initial value and after 30 d of olive oil or lycopene-olive oil intake.

	Initial	1 Month	
		Lycopene-Olive Oil (%) *	Olive Oil (%) *
CRP (mg/L)	100 ^a^	85 ± 19 ^b^	94 ± 24 ^a^
sCD40-L (ng/L)	100 ^a^	111 ± 46 ^a^	133 ± 40 ^b^
IL-6 (pg/mL)	100 ^a^	88 ± 36 ^b^	116 ± 30 ^a^

* Percentage of initial value. Values are means ± SD. Values with different alphabetical letters are significantly different (*p* < 0.005). RCP = C-Reactive Protein, IL-6 = interleukin 6.

## Data Availability

The data are contained within the article and supplementary materials.

## Supplementary Materials

The following supporting information can be downloaded at: https://www.mdpi.com/article/10.3390/antiox12071458/s1, Table S1: Composition (%) of to3mato extract-olive oil in each container of 20 mL; Table S2: Characteristics of the study subjects at the beginning of study.
