# Supplementary material for: Beneficial Effects of Olive Oil Enriched with Lycopene on the Plasma Antioxidant and Anti-Inflammatory Profile of Hypercholesterolemic Patients
Source: Antioxidants (Basel). 2023 Jul 20;12(7):1458. doi: 10.3390/antiox12071458 (PMC10376681; doi:10.3390/antiox12071458)
Supplement: Supplementary file 1 [file antioxidants-12-01458-s001.zip › antioxidants-2441283-SI.pdf]

**Supplemented 1. Composition (%) of to3mato extract-olive oil in each container of 20 mL**

| | Olive oil-tomato extract |
|---|---|
| Lycopene (%) | 0.05 |
| Beta-carotene (%) | 0.005 |
| Phytosterols (%) | 0.16 |
| Oleic acid (%) | 71.62 |
| Tocopherols (%) | 0.04 |
| Phytoene/phytofluene (%) | 0.01 |
| Tomatoes oil (%) | 0.58 |
| Linoleic acid (%) | 9.29 |
| Linolenic acid (%) | 1.07 |
| Gadoleic acid (%) | 0.26 |
| Palmitoleic acid (%) | 1.03 |
| Palmitic acid (%) | 11.9 |
| Margaroleic (%) | 0.14 |
| Arachidic acid (%) | 0.47 |
| Phospholipids (%) | 0.07 |
| Stearic acid (%) | 3.24 |
| Antioxidant capacity | |
| ABTS (mM Trolox) | 3.21 ± 0.032 |
| DPPH (mM Trolox) | 670 ± 68 |
| FRAP (mM) | 0.826 ± 0.05 |
| Antiradical capacity | |
| HRSA (% inhibition) | 91.1 ± 0.90 |
| SRSA (% inhibition) | 42.6 ±10.1 |

**Supplemented 2. Characteristics of the study subjects at the beginning of study**

| | Females (n=25) | Males (n=55) |
|---|---|---|
| Age (y) | 58±13 | 61± 12 |
| Energy (Kcal) | 1865 ± 442 | 2013 ± 168 |
| Carbohydrates (g) | 166 ± 54 | 168 ± 45 |
| Protein (g) | 65 ± 20 | 97 ± 20 |
| Fiber (g) | 17.8 ± 6.8 | 15.7 ± 4.7 |
| Cholesterol (mg) | 371 ± 109 | 377 ± 90 |
| Fatty acids | | |
| SFA | 33.7 ± 23.3 | 26.3 ± 8.3 |
| MUFAs | 50.6 ± 32.9 | 45.4 ± 11.8 |
| PUFAs | 15.8 ± 13.4 | 12.2 ± 3.8 |
| Vitamin B12 (µg) | 5.7 ± 2.1 | 6.2 ± 2.2 |
| Vitamin C (mg) | 148.2 ± 71.9 | 109.3 ± 41.8 |
| Vitamin D (µg) | 10.4 ± 10.7 | 4.07 ± 5.55 |
| Folic acid (µg) | 508 ± 276 | 240 ± 107 |
| Calcium (mg) | 848 ± 288 | 753 ± 287 |
| Magnesium (mg) | 273 ± 73 | 261 ± 71 |
| Fe (mg) | 12.4 ± 3.8 | 12.3 ± 3.0 |
